# 

**Published:** 1902-04-15

**Authors:** 


					﻿THE
DENTAL REGISTER
EDITED BY
NELVILLE S. HOFF, D. D. S.,
ANN ARBOR, MICH.
Vol. LVI.	APRIL 15, 1902.	No. 4.
PRICE, ONE DOLLAR A YF AR.LN.JlDuVANG&r
PUBLISHED MONTHLY BY
SAMUEL· A. CROCKER & CO.,
THE OHIO DENTAL AND SURGICAL DEPOT.
to 3$) W. “tli S^t., Ciiieinniiti, O.
Entered at the Postoffice at Cincinnati, 0., as second-class mail matter.
				

